# Women's Knowledge, Attitude, and Perceptions Toward COVID-19 in Lower-Middle-Income Countries: A Representative Cross-Sectional Study in Bangladesh

**DOI:** 10.3389/fpubh.2020.571689

**Published:** 2020-11-17

**Authors:** Saeed Anwar, Yusha Araf, Asir Newaz Khan, Md. Asad Ullah, Nur Hoque, Bishajit Sarkar, Riyan Al Islam Reshad, Rahatul Islam, Nurshad Ali, Mohammad Jakir Hosen

**Affiliations:** ^1^Department of Medical Genetics, Faculty of Medicine & Dentistry, University of Alberta, Edmonton, AB, Canada; ^2^Department of Genetic Engineering and Biotechnology, School of Life Sciences, Shahjalal University of Science and Technology, Sylhet, Bangladesh; ^3^Department of Economics and Social Sciences, Brac University, Dhaka, Bangladesh; ^4^Department of Biotechnology and Genetic Engineering, Faculty of Biological Sciences, Jahangirnagar University, Dhaka, Bangladesh; ^5^Department of Statistics, Faculty of Science, University of Dhaka, Dhaka, Bangladesh; ^6^Department of Biochemistry and Molecular Biology, School of Life Sciences, Shahjalal University of Science and Technology, Sylhet, Bangladesh

**Keywords:** COVID-19, SARS-CoV-2, women, health literacy, knowledge, awareness, preparedness, attitude

## Abstract

The coronavirus disease 2019 (COVID-19) is a global health emergency of unprecedented proportions. Countries around the world have taken extraordinary steps to control the disease. The preventive measures face challenges in low and lower middle income countries (LICs and LMICs). Especially the marginalized communities, e.g., women are the hardest hit of the virus. This study took Bangladesh as a representative LMIC and aimed to determine the level of knowledge, perception, attitude, and preparedness related to COVID-19 among the adult women in the country. Using a comprehensive questionnaire, we channeled a cross-sectional study among adult women in Bangladesh. Participant's self-reported data on the knowledge, attitude, and preparedness were tabulated and analyzed using suitable statistical tools. A total of 1,869 adults from 61 districts of Bangladesh took part in this study. Ninety seven percentage of the participants claimed to have heard of COVID-19 before it arrived in Bangladesh. Regarding the general knowledge related to COVID-19's causal agent, symptoms, and treatment, the positive response rate was nearly 80%, with a mean of 10.68 ± 1.72. Younger and educated women had better knowledge levels compared to the older and lower-educated participants (*p* < 0.01). More efforts are required to educate women with older age and lower socioeconomic status. An overall positive attitude and perception were observed, although a significant proportion of the participants opined that the Government's efforts in controlling the outbreak were not adequate. Although the participants had a satisfactory level of knowledge and a positive attitude in adopting preventive measures against COVID-19, greater efforts are needed from the healthcare authorities and Government.

## Introduction

The severe acute respiratory syndrome coronavirus 2 (SARS-CoV-2) is a new member of the coronaviridae family of RNA viruses (1). Infection with SARS-COV-2, leading to coronavirus disease 2019 (COVID-19) in humans, can result in respiratory syndromes ranging from an uncomplicated upper respiratory tract distress to severe viral pneumonia with multiorgan failure and death (2). This new virus transmits by droplets from asymptomatic or oligosymptomatic patients and proximally through aerosols in health care environments (3, 4). Within months after the first infection detected in humans late in 2019, this highly contagious virus with the ability to cause severe respiratory disease has hit the health systems across the world (5). In around 6 months after the first emergence of the virus, nearly 4.5 million confirmed cases have been identified in 185 countries around the globe, and over 300 thousand people have died of the disease (6).

The disease has evolved into a pandemic, and the World Health Organization (WHO) has declared it a global health emergency of unprecedented proportions (7). The outbreak substantially impacted millions of people around the world. As there is yet a vaccine or treatment strategy to be approved for COVID-19, only strong infection control measures can help minimize the spread of the virus in the community and health care facilities. Countries worldwide adopted extraordinary control measures soon after the virus's emergence, and a multi-level stress-coping-adjustment procedure is in progress (8). In order to execute the control measures effectively, every individual across the world requires to pay attention to the dramatically changing messages about public health and take prompt actions to limit the virus spread and individual risk (7, 8). Lessons learned from previous outbreaks indicate that poor knowledge, attitudes, and perception (KAP) toward infectious diseases and health literacy may challenge the efforts to prevent the spread of disease (9). Besides, under-estimating potential risk, stigmatization, panic emotions, and wrong measures to avoid the infection may affect combating such a situation (10).

However, the prevailing rhetoric related to the pandemic is often not intelligible and evenly disseminated to the mass people (11). Consequently, the messaging regarding the strategic measures and imminent threat of COVID-19 pandemic is at odds, leading to miscommunication, public confusion, and inaction (12). The situation is far more complicated in low and lower-middle-income countries (LICs and LMICs), like in Bangladesh, where significant portions of the population have minimal health literacy (13, 14). Among the people in these underprivileged communities, women are even more disadvantaged due to cultural norms and values. A large proportion of women in these countries have negligible access to information (15). As a result, women in LICs and LMICs supposedly have lower knowledge-index. In the situation of a health emergency, when knowing and understanding the critical and rapidly changing health messages come with foremost importance, people living in marginalized communities, like the women in LICs and LMICs with limited access to information, may experience extremely inadequate health communication (16, 17). The limitations of health communication can potentially lead these people to be further marginalized and exposed to elevated risks (17, 18).

We did a time-sensitive study among the adult women representing different social groups of Bangladesh, an LMIC in Southeast Asia. Non-therapeutic preventive measures to limit the spread of SARS-CoV-2, e.g., social distancing, faced an enormous challenge during the early stage of the outbreak in Bangladesh. With a huge lack of diagnostic and hospital facilities and poor coordination of management strategies, the country was walloped by the emergence of the virus (19). Assumably, health literacy, and awareness among the mass people, especially among the marginalized population like the women, would be vital to control the virus spread and mitigate the pandemic's impact. This study aimed to assess knowledge, attitudes, and perceptions about COVID-19 among the female population in a resource-limited LMIC.

## Methods

### Study Design and Population

This cross-sectional survey took place during the initial weeks of lockdown enforced in Bangladesh. Only adult women of Bangladeshi nationality, who are not working in the medical field, were recruited in the study using convenience and snowball sampling methods. Given the current situation, a thorough community-based survey was not feasible. We collected the data through telephone, online, or in-person interviews, when possible. A team comprised of graduate students who majored in health and life sciences, clinicians, and statisticians conducted the questionnaire-based interviews.

### Measurements

The newly prepared questionnaire consisted of five parts concerning the demographic backgrounds of the participants, and their knowledge, attitude, and preparedness (KAP) related to COVID-19. Following the guidelines for clinical and community management of COVID-19 by authorized bodies, we developed this questionnaire to assess the self-reported KAP of the participants (7, 20–22). The questionnaire included 15, 25, and 17 items related to the knowledge, attitude/perception, and preparedness of the women related to COVID-19. Each positive response (correct answer, where applicable) in the knowledge section was given 1 point, whereas a negative response received a 0 point.

### Validation of the Questionnaire

We evaluated the newly prepared questionnaire in a preliminary study. Initially, we asked a group of Bangladeshi researchers in the field of epidemiology to independently assess the degree to which the questionnaire is relevant and is able to measure women's KAP regarding COVID-19 correctly. In terms of language, formatting, and contents, we made essential modifications to the questionnaire to address their comments. Later on, to pre-test the questionnaire, we interviewed 35 participants twice 15 days apart using the modified questionnaire. Obtained data were used to assess internal consistency and test-retest reliabilities using Cronbach's α and intra-class correlation analysis. Both assessments indicated satisfactory levels of reliability of the questionnaire (Cronbach's α = 0.79, intra-class correlation coefficient = 0.96). No data from the above mentioned 35 participants were included in the final analyses.

### Statistical Analysis

We evaluated all data using the Statistical Package for the Social Sciences (IBM SPSS, v 22.0; Chicago, IL) software (23). Associations between participant characteristics and survey responses were then examined in bivariate analyses using Student's *t*-tests, z-statistic, χ2 tests, or analysis of variance (ANOVA), as suitable. For the continuous outcome of a perceived concern, we used multivariable linear regression models to estimate least-squares means (with 95% confidence intervals). The quantitative variables were reported either as mean ± standard deviation or frequency (%). All computations included the KAP variables as primary covariates of interest. Other variables affecting the KAP were also analyzed using appropriate statistical tools. All analyses were performed at α-levels of 0.05 and 0.01 (*p* = 0.05 and *p* = 0.01, respectively).

### Ethics Statement

The internal Ethical Review Board (ERB) at the Department of Biochemistry and Molecular Biology, Shahjalal University of Science and Technology approved the study protocol (Reference ID: 02/BMB/2020). All participants in this study provided informed consent as per the World Medical Association (Helsingin julistus). No participants received any monetary rewards for participating in the study.

## Results

### Participants' Characteristics

In total, our interviewers invited 3,150 adult women to participate in this study, of which 1,246 declined to participate (Supplementary Table 1). A total of 1,904 women took part in the interviews; however, we excluded questionnaires of 35 participants due to incompleteness. Henceforth, the final analysis of this study consisted of data obtained from 1869 women, giving an overall response rate of 59.33%.

The mean age of the participants was 29.545 ± 12.009 years (range: 18–86 years), with over 40% (43.23%) of them aged 18 to 30 years and only 5.67% aged 60 years and above (Table 1, Supplementary Figure 1). The sample pool consisted of diverse socioeconomic backgrounds, originating from 61 of 64 districts of Bangladesh (Table 1, Supplementary Figures 1, 2). Over 50% (51.20%) of the participants had higher secondary or more education, with 17.39% of them being university or college graduates. Nearly one-third of the participants (32.69%) were students, another one-third (37.72%) were either unemployed, retired, or housewives (not involved in earning). Besides, ~60% (61.10%) of participants were either single or ever married at the time of the study. In terms of religious background, over 86% (86.35%) of participants were Muslims (Table 1). Over 10% (12.09%) of all participants had multiple clinical conditions (>1 clinical conditions), including coronary complications, respiratory, and pulmonological complications (Supplementary Table 2). Nearly 10% (9.84%) of the participants reported that they frequently experienced feverish symptoms.

**Table 1:** Sociodemographic characteristics of participants.

**Variables**	**Value (*n* = 1,869)**
Age (years)	29.545 ± 12.009
**Marital status (*****n*****, %)**	
Single	801, 42.86
Married	727, 38.90
Ever married	341, 18.24
**Education level (*****n*****, %)**	
No schooling	88, 4.71
Primary	240, 18.84
Secondary	508, 27.18
Higher secondary	632, 33.81
University/college	325, 17.39
Other	76, 4.07
**Occupation (*****n*****, %)**	
Unemployed/retired	294, 15.73
Student	611, 32.69
Self-employed	43, 2.30
Business	102, 5.45
Maidservants/household helping hands	146, 7.81
Service holder in a Government organization	105, 5.61
Service holder in a non-government/private organization	157, 8.40
Housewives (not involved in earning)	411, 21.99
**Religion (*****n*****, %)**	
Islam	1,614, 86.35
Hindu	214, 11.44
Buddhist	27, 1.44
Christian	05, 0.27
Prefer not to say	09, 0.48
**Ethnicity (*****n*****, %)**	
Bengali	1,848, 98.88
First nations/tribal	21, 1.12

### Knowledge Related to COVID-19

97% (*n* = 1,813) of all participants reported having heard of the COVID-19 outbreak. Out of the 1,813 participants who had heard of the outbreak, 1,759 (97.02%) knew that the virus had arrived in Bangladesh (Table 2). The source of knowledge for most the participant was internet (*n* = 1,173, 64.7%) and the TV (*n* = 919, 50.69%) (Supplementary Table 3). Over two-thirds (67.84%) of the participants claimed that they knew about COVID-19 after its emergence in China and before it arrived in Bangladesh (Supplementary Figure 3). Overall, the positive response rate was nearly 80% (Table 2). The total knowledge score ranged from 4 to 14, with a mean of 10.68 ± 1.72.

**Table 2:** Women's knowledge related to COVID-19.

	**Response**		
	**Yes *(n*, %)**	**No (*n*, %)**	**Maybe (*n*, %)**
Knows about contagious diseases	1,616, 86.46	178, 9.52	75, 4.01
Knows about viral flus	1,573, 84.16	231, 12.36	65, 3.48
Has idea about the general flu protocol of WHO	1,248, 66.77	450, 24.08	171, 9.15
Knows what causes (causal agent) COVID-19	1,529, 81.81	256, 13.7	84, 4.49
Knows that COVID-19 is a contagious disease	1,424, 76.19	331, 17.71	114, 6.1
Knows about the mode of transmission of COVID-19	1,506, 80.58	265, 14.18	98, 5.24
Knows about the symptoms of COVID-19	1,440, 77.05	315, 16.85	114, 6.1
Knows about the unavailability of COVID-19 treatments	1,501, 80.31	267, 14.29	101, 5.4
Knows who are the vulnerable group to COVID-19	1,488, 79.61	276, 14.77	105, 5.62
Knows what quarantine means	1,392, 74.48	359, 19.21	118, 6.31
Knows what social distancing means	1,424, 76.2	332, 17.76	113, 6.04
Overall response rate (mean ± SD)	78.51 ± 5.06	15.86 ± 3.7	5.63 ± 1.42

Table 2, Supplementary Figure 4 presents the outcomes of knowledge assessment of the women regarding COVID-19's mode of transmission, common symptoms, vulnerable groups, and rhetoric related to the pandemic.

### Perceptions and Attitudes Related to COVID-19

Nearly 4 out of 5 (83.28%) women acknowledged that they fear COVID-19 (Table 3). Over 50% (52.56%) of the participants were concerned because they have older family members. Around 3 out of 4 participants perceived COVID-19 as a dangerous public health threat (75.46%). However, they thought it to be common cases of flu (72.59%) (Table 3). One-fourth of the women (25.32%) assumed that COVID-19 is a curse from the GOD. Only ~40% of women thought that people around them are aware of the COVID-19 situation. The majority of the participants responded that Bangladesh's efforts and preparations in COVID-19 management was not enough and satisfying (Table 3). 46.8% thought that the media coverage about this disease is exaggerated. Two out of five women (39.33%) thought that the Bangladesh Government's timely measures could help reduce the spread of the virus, while only one-third (34.80%) thought that the doctors and nurses in Bangladesh are trained to handle COVID-19 patients. Over 85% (87.42%) opined that the Government should subsidize for treatment of COVID-19 patients. More than a half (53.94%) thought that the Government was not transparent on COVID-19 related information. Nearly half (46.39%) of the women feared that COVID-19 would result in a devastating fatality in the country (Table 3).

**Table 3:** Perceptions of the women about COVID-19.

**Perceptions and attitudes about COVID-19**	**Response (*****n*** **=** **1,813)**		
	**Yes (*n*, %)**	**No (*n*, %)**	**Maybe (*n*, %)**
I fear COVID-19	1,510, 83.29	179, 9.87	124, 6.84
I am scared because my family have older (>60 yrs) adults (including me)	953, 52.56	261, 14.4	599, 33.0
COVID-19 is like the common-flus	1,316, 72.58	348, 19.19	149, 8.22
COVID-19 is a dangerous public health threat	1,368, 75.46	249, 13.73	196, 10.81
I am satisfied with Bangladesh's efforts to tackle the pandemic	849, 46.83	760, 41.92	204, 11.25
COVID-19 arrived in Bangladesh by people coming from abroad	1,227, 67.68	311, 17.15	275, 15.17
COVID-19 is a religious curse	459, 25.32	1090, 60.12	264, 14.56
Aggressive screening would help the management of COVID-19	1,556, 85.82	158, 8.71	99, 5.46
Bangladesh have enough facilities for screening COVID-19	683, 37.67	743, 40.98	387, 21.35
People around you are aware of the current situation	726, 40.04	928, 51.19	159, 8.77
Bangladesh have enough ventilation facilities to help critical patients	629, 34.69	853, 47.05	331, 18.26
Timely measures by the Government could help reduce the spread of COVID-19 in Bangladesh	771, 42.53	713, 39.33	329, 18.15
The government should subsidize for treatment of COVID-19	1,585, 87.42	129, 7.16	99, 5.46
Bangladeshi doctors and nurses are trained to treat COVID-19 patients	631, 34.80	826, 45.56	356, 19.64
The mosques and religious congregations should remain discontinued	845, 46.61	742, 40.93	226, 12.47
Bangladesh is economically able to tackle COVID-19 challenge	507, 27.96	1193, 65.80	113, 6.23
COVID-19 pandemic may cause a food crisis in the country	852, 46.99	813, 44.84	148, 8.16
Hand sanitizers, hand soaps, and masks should be available freely	1,127, 62.16	556, 30.67	130, 7.17
Bangladeshi doctors have enough personal protective equipment	919, 50.69	771, 42.53	123, 6.78
The pandemic will severely hamper the education system	1,316, 72.59	354, 19.58	142, 7.83
COVID-19 will cause devastating fatality in Bangladesh	841, 46.39	631, 34.80	341, 18.81
Media coverage about this disease is exaggerated	848, 46.77	649, 35.8	316, 17.43
The government is transparent on COVID-19 information in Bangladesh	609, 33.59	978, 53.94	226, 12.47
Bangladesh is dependent on foreign grants for controlling COVID-19	763, 42.08	825, 45.5	225, 12.41
Bangladesh will collapse due to COVID-19 pandemic	786, 43.35	753, 41.53	274, 15.11

### Preparedness Related to COVID-19

**Figure 1:**
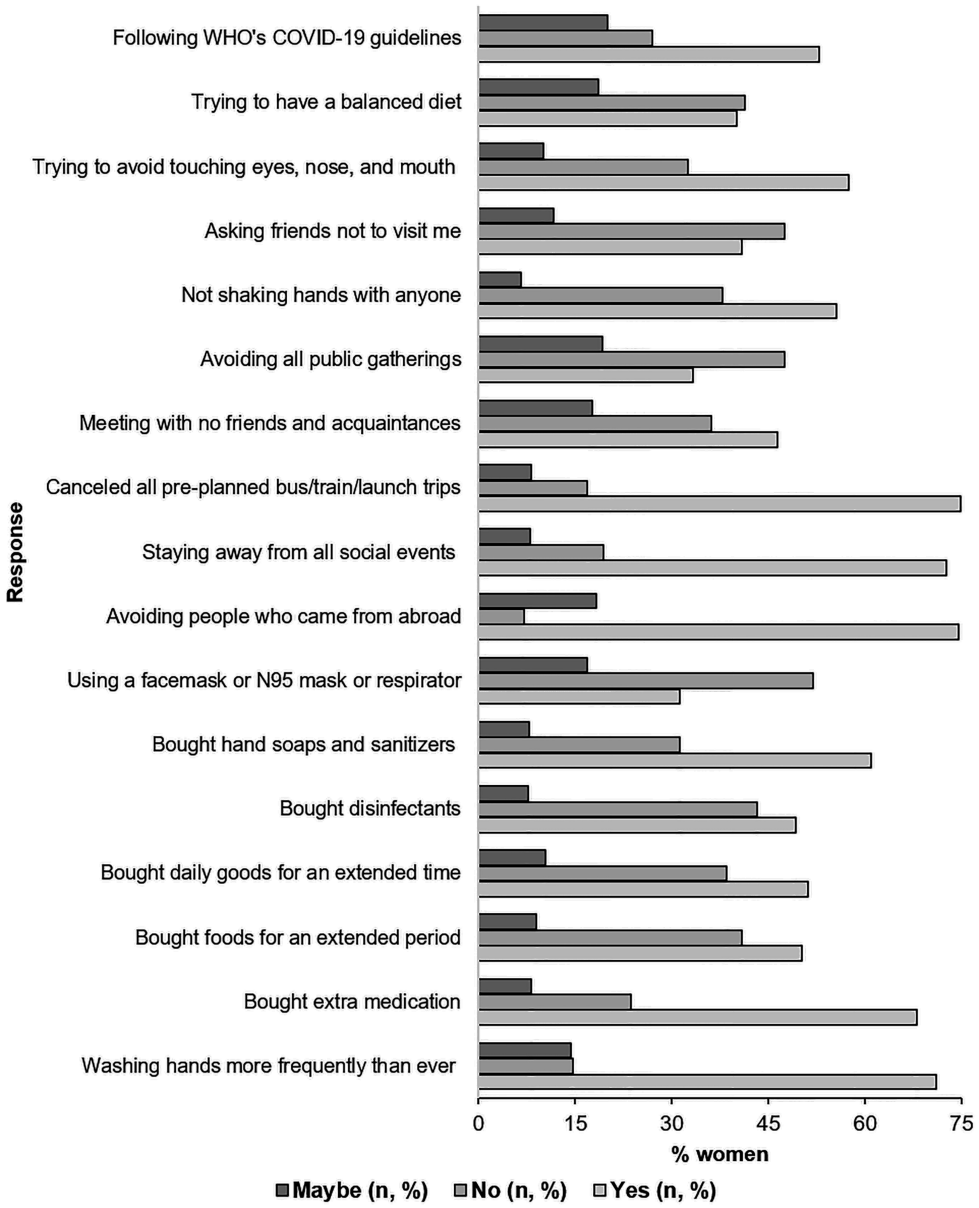
Womens preparedness related to COVID-19.

Figure 1 presents the preparedness of the women to limit the spread of COVID-19 and their responses. Seventy one percentage of participants reported that they are washing their hands more frequently than ever and for an extended period (Figure 1). They also reported that they managed to buy extra foods (50.27%), medicines (68.16%), daily goods (51.16%) for an extended period, and also bought disinfectants and hand soaps/sanitizers as precautions (48.66% and 61%, respectively) (Figure 1). Two out of five of every woman who participated claimed that they were avoiding to meet with their friends even if they have no symptoms (46.51%), to attend any public gatherings (e.g., political or religious gatherings) (38.82%) and places where many people used to gather (42.21%). Over 70% (74.59%) of the participants also claimed to abstain from meeting with anyone who has recently come from abroad. Nearly 60% (58.49%) of the women reported that they were trying to follow the guidelines of WHO regarding COVID-19. Around 40% of the participants (40.07%) women were using either a facemask or a KN95 mask/respirator (Figure 1).

### Sociodemographic Characteristics and COVID-19 Related Knowledge

We observed a significantly different mean knowledge scores between different age groups (*p* < 0.01) (Table 4). Compared to the younger age groups, participants aged 51–60 years and >60 years had a significantly lower mean knowledge score (9.70 ± 1.6 and 9.95 ± 1.99, respectively). Also, women from urban areas had a significantly higher mean knowledge score (10.87 ± 1.65) compared to residents from rural areas (10.01 ± 1.79) (*p* < 0.01). The knowledge mean scores did not correlate significantly to the education-levels of the women (*p* = 0.001). Also, women who did not participate in active earning (unemployed/retired and housewives) had a significantly lower knowledge level (*p* = 0.0018).

**Table 4:** Factors affecting the knowledge score of women.

**Predictor**	**Knowledge score (mean, SD)**	***p*-value**
**Age (years)**		
18–30	10.89 ± 1.75	< 0.001
31–40	10.8 ± 1.64	
41–50	10.82 ± 1.76	
51–60	9.7 ± 1.6	
>60	9.95 ± 1.99	
**Education**		
High school or below	10.61 ± 1.71	0.001
College/university or above	10.93 ± 1.74	
**Marital status**		
Single	10.77 ± 1.7	
Married	10.62 ± 1.78	
Ever married	10.59 ± 1.63	
**Area of residence**		
Urban	10.87 ± 1.65	<0.001
Rural	10.01 ± 1.79	
Slum	9.68 ± 1.7	
**Occupation**		
Student	11.01 ± 1.8	0.1249
Involved in active earning	10.86 ± 1.5	
Unemployed/retired and housewives	10.56 ± 1.82	0.0018

* *SD, standard deviation*.

## Discussion

With one of the world's densest populations, Bangladesh is an LMIC in Southeast Asia. Like most other LICs and LMICs, it has limited health facilities, and its citizens' health literacy level is not satisfactory (24, 25). As a result, it supposedly faces a significant challenge in implementing any health measures. Besides, many senior citizens and mid-aged people in the country have non-communicable disorders, including chronic obstructive pulmonary disease (11.9%), cardiac disorders (4.5%), diabetes (9.7%), and asthma (5.2%) (26–29). These people with multiple comorbidities are also especially vulnerable to emerging infectious diseases, e.g., the COVID-19.

As the COVID-19 outbreak quickly surges around the globe, every country is taking extraordinary measures to control its spread. Since there is yet a vaccination or effective treatment strategy against the disease, the control measures basically include non-therapeutic preventive strategies. Effective implementation of these measures requires wholehearted efforts by the government bodies, together with personal understanding and practices, and it depends on the KAP of the general public about the disease. Usually, in the LICs and LMICs, general people, especially the marginalized communities like the females, are less aware of health-related issues. The health-related marginalization of women is due to the availability of few recourses to access health service and literacy. As a result, they show poor perception and attitude toward health issues. While combating a global health crisis like the COVID-19 pandemic, the poor KAP of women may hamper the implementation of control and mitigation strategies. They remain at the core of the fight against the COVID-19 pandemic (30). It is also reported that women, especially the women workers, are among the hardest-hit groups by COVID-19 in Bangladesh (31). Here we present the results of a survey about the KAP of the Bangladeshi women toward the COVID-19 disease. It represents the first study to evaluate the KAP-level among women in LICs and LMICs, including Bangladesh.

The educated women, having an equivalent or higher education than higher-secondary level (51.2%), dominated the study population (Table 1). Also, a significant proportion of the women were students, preferably of college or university levels (Table 1). From the perspective of religious background, ethnicity, the structure of the studied population was comparable with the general female population of Bangladesh (Table 1) (32). Interestingly, the prevalence of comorbid conditions was relatively high compared to the country's overall frequency (26–29). Nearly 40% of the women (40.67%), among who were invited to participate, declined to participate (Supplementary Table 1). The reason for declining to participate included no time for participating (60.67%), lack of interest (29.13%), and fear of forgery (11.56%). The final sample included data from 1,869 women, representing an ideal survey sample size (at a 99% confidence interval, the limit of precision of 1%) (Supplementary Table 1). Overall, though educated women dominated the sample population, it was diverse and corresponded to the general trends of Bangladesh's population. However, as we distributed the survey using convenience and snowball methods, it may have influenced the general characteristics of the sample population. Given the overall situation due to COVID-19, we were unable to conduct a more rigorous survey, and this represents a major limitation of this study.

In general, women who participated in our study had good general knowledge about the disease, its mode of transmission, and prevention (Table 2). The primary sources of knowledge on COVID-19 among women included the internet (64.7%) and TV (50.69%) (Supplementary Table 2). A possible reason for the internet being the most favored source of knowledge could be the inclusion of more young women, e.g., students who used to surf the internet more than the mid-aged and older women. These channels of knowledge, e.g., the internet and TV, provide an uncomplicated and accessible way to receive information related to COVID-19; these can also provide misinformation, fabricated data, and rumors (33, 34). Henceforth, caution about the use of these channels must be in place (34–36). The overall response rate was nearing 80% (78.51 ± 5.06)%, and the average knowledge score was 10.68/16 (±1.72), denoting a satisfactory level of knowledge on COVID-19's causal agent, mode of transmission, symptoms, vulnerability group, relevant rhetorics, and treatment. Women's COVID-19 related knowledge level observed in this study exceeded the knowledge levels of both general (includes both men and women) and female sub-groups reported in two other recent studies held in Bangladesh (37, 38). Given that both of these studies report the outcomes based on sample populations dominated by young and educated individuals, it indicates that women in Bangladesh are more knowledgeable about COVID-19 than men.

We observed a comparatively lower COVID-19 related knowledge level among older women (Supplementary Figure 1). These results are similar to the outcomes reported for the general population of Bangladesh, Egypt, and China, where participants with high socioeconomic status were more knowledgeable than participants coming with lower status (10, 35, 37, 38). It indicates that extensive efforts are required to deliver messages to the older group of women, who may have difficulties accessing the most favorable sources of COVID-19 related knowledge.

When analyzing women's perception and attitude toward COVID-19, we found that over four of five women were scared of the infection, and the primary reason (52.56%) for their fear was due to the presence of older individuals in their family (Table 3). A similar response was reported from other studies held on general populations as well (10, 35). Unlike previous reports in other LMICs, around half of the participants assumed that the media was exaggerating the risk (Table 3) (35, 39).

The majority of the women opined that the Bangladeshi administration and health professionals had inadequate preparations to combat the outbreak. Many of the women were also concerned about the awareness of the people around them. It is apparent in the participants' opinion that Bangladeshi authorities failed to manage the COVID-19 outbreak better, which could lead to a devastating fatality in the country. Bangladesh's poor preparation for tackling the COVID-19 situation was also reported in a recent interagency memo of the United Nations, led by the WHO (40). Although most of the participants were concerned about a possible economic crisis, they opined that the Government should subsidize the treatment of COVID-19. Concerns over the Government's transparency regarding COVID-19 related information, suppressing unrestricted dissents, the possibility of food unavailability in the future, and education of children (and themselves) were also in place. Many were scared that the country would collapse in tackling the pandemic. However, the authorities of Bangladesh have repeatedly claimed that their prompt measures kept the outbreak of COVID-19 well under control in the country, and the Government is fully transparent about its policies to mitigate the situation (41–44).

One-fourth of our participants assumed the COVID-19 as a religious curse. In response to the extraordinary situations due to COVID-19, many countries curbed the religious congregations, and some Muslim-dominant countries even temporarily amended the adhan (call for prayer) to urge followers to pray in their homes than to come to the mosques (45). Bangladesh also suspended regular and special prayers in the mosques and applied restrictions to all other religious groups (46). Interestingly, a mixed opinion was observed regarding public religious congregations among the women in Bangladesh (Table 3). A possible reason for this mixed opinion could be the strong influence of religious beliefs among the population of semi-conservative societal structure in Bangladesh- where people used to face dilemmas in amending religious practices even when there are logical grounds (47).

Participants in our study showed a sort of good personal preparedness in response to COVID-19 (Figure 1). They considered the value of frequent handwashing for an extended time, avoiding to touch eyes, nose, and mouth, and limited personal contact. However, the proportion of women was low compared to the general population (internet users), as described by previous studies held in Bangladesh, where ~95% of participants were practicing social distancing (37). In our study, ~75% of the participants avoided social gatherings, meeting with people coming from abroad, and canceled preplanned visits during the summer vacations. Nearly half of the women stocked more foods, daily goods, and regularly needed medicines for a longer time. Also, many of them bought hand soaps, sanitizers, and disinfectants. Around 40% of women were using a facemask or KN95 mask or a respirator. The percentage of participants practicing the use of masks was comparable with the reports from Egypt, but not with China, where almost all participants reported putting face masks when they go out (10, 35). It is apparent that the use of masks was substantially low among women in Bangladesh as compared to the internet users (men and women) in the country, >91% of whom used to put a mask when going outside the home (37). Regarding the use of masks, the Center for Disease Control and Prevention (CDC) emphasized putting face coverings in areas where the transmission is at the community level, while WHO recommends using it for those who have respiratory symptoms or are caring for another person with symptoms (48, 49). It is crucial to develop local guidelines for mask-use in Bangladesh by the health experts and Government bodies. Unnecessary use of masks needs to be prevented while confirming that the health risk is not hampered during this unprecedented time. Similar guidelines are also needed for the use of hand soaps, sanitizers, and disinfectants, as overuse of these chemical substances may harm the dermatological aspects of men.

Bangladesh's efforts in controlling the COVID-19 pandemic had numerous limitations (19). As the country had, like almost all other countries, no experience in facing a coronavirus outbreak, weakness in its preparation is relatable. Besides, as a small country with limited resources but a huge population, its efforts faced enormous challenges (19). The KAP of its citizens, including marginalized communities like women, was satisfactory at individual levels, which is essential to control the infection (9, 10, 50). However, the country's efforts seem to fail in controlling the spread (37, 38). As of early October, 6 months after the detection of the first COVID-19 case in Bangladesh, the number of active and new cases is increasing by leaps and bounds (51). A similar situation is observed in other LMICs in Bangladesh's neighborhood and beyond (39, 52–54). Although the Governments of the countries have taken significant measures to limit the spread of the disease, more effort is needed to support the most affected groups.

This study indicates that the healthcare authorities, media, and Governments in LICs and LMICs should be more careful and transparent in spreading knowledge to its people, especially to those who are marginalized, e.g., women and older individuals with low socioeconomic status, when fighting a pandemic like the COVID-19.

## Data Availability Statement

All datasets generated for this study are included in the article/Supplementary Material.

## Ethics Statement

The studies involving human participants were reviewed and approved by Ethical Review Board (ERB), Department of Biochemistry and Molecular Biology, School of Life Sciences, Shahjalal University of Science and Technology, Sylhet - 3114, Bangladesh (Reference ID: ERB/02/BMB/2020). The patients/participants provided their informed consent to participate in this study.

## Author Contributions

MH conceived the study. MH, SA, YA, and NA designed the study. MU, YA, BS, RR, RI, and NA conducted the surveys. SA analyzed and interpreted the data. AN and NH helped the interpretation of the data. SA and MU wrote the draft manuscript. SA and NA carried out the revisions. All authors approved the final version of the manuscript.

## Conflict of Interest

The authors declare that the research was conducted in the absence of any commercial or financial relationships that could be construed as a potential conflict of interest.
