# Supplementary figures and images for: Women's Knowledge, Attitude, and Perceptions Toward COVID-19 in Lower-Middle-Income Countries: A Representative Cross-Sectional Study in Bangladesh
Source: Front Public Health. 2020 Nov 17;8:571689. doi: 10.3389/fpubh.2020.571689 (PMC7707120; doi:10.3389/fpubh.2020.571689)

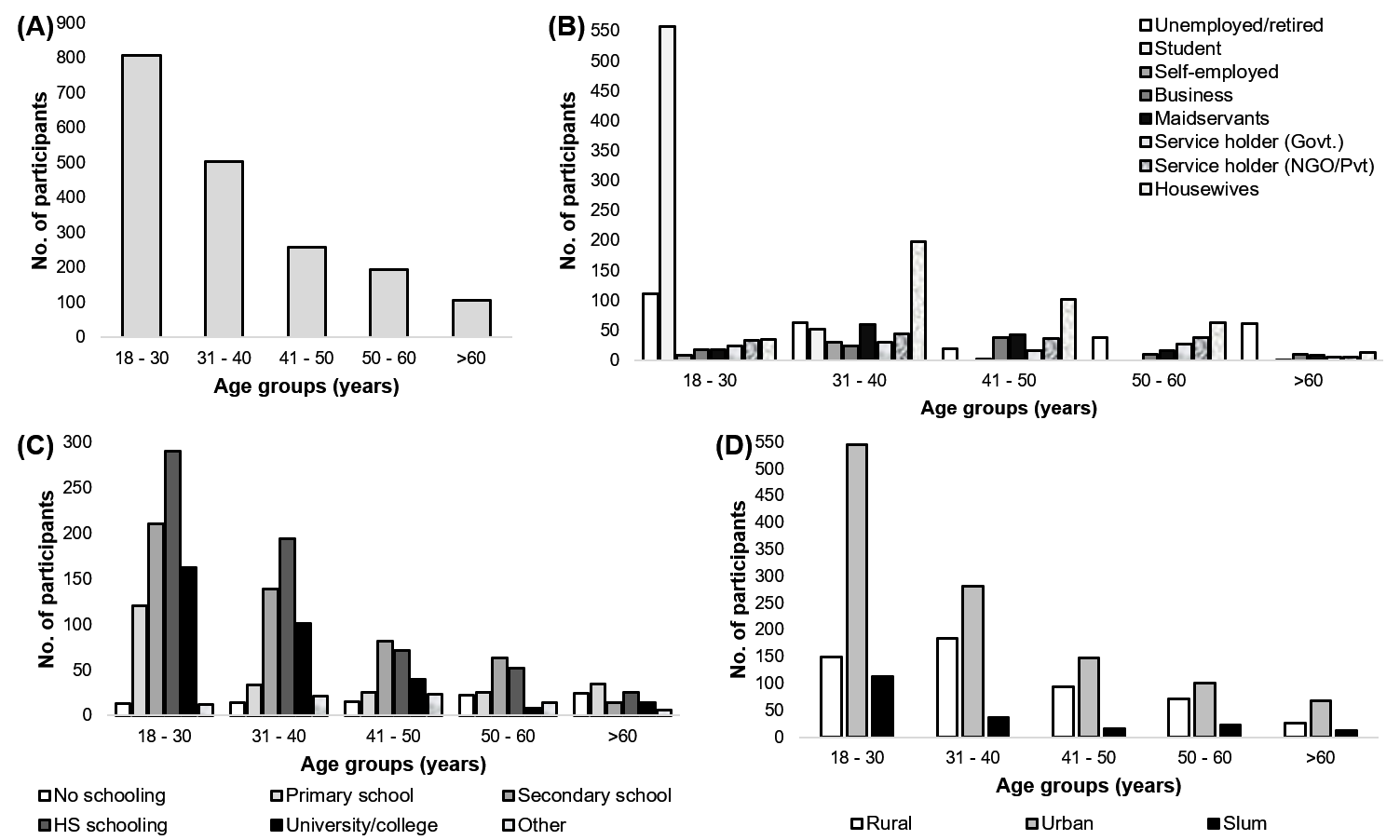

Supplement: Supplementary file 1 [file Image_1.TIF]

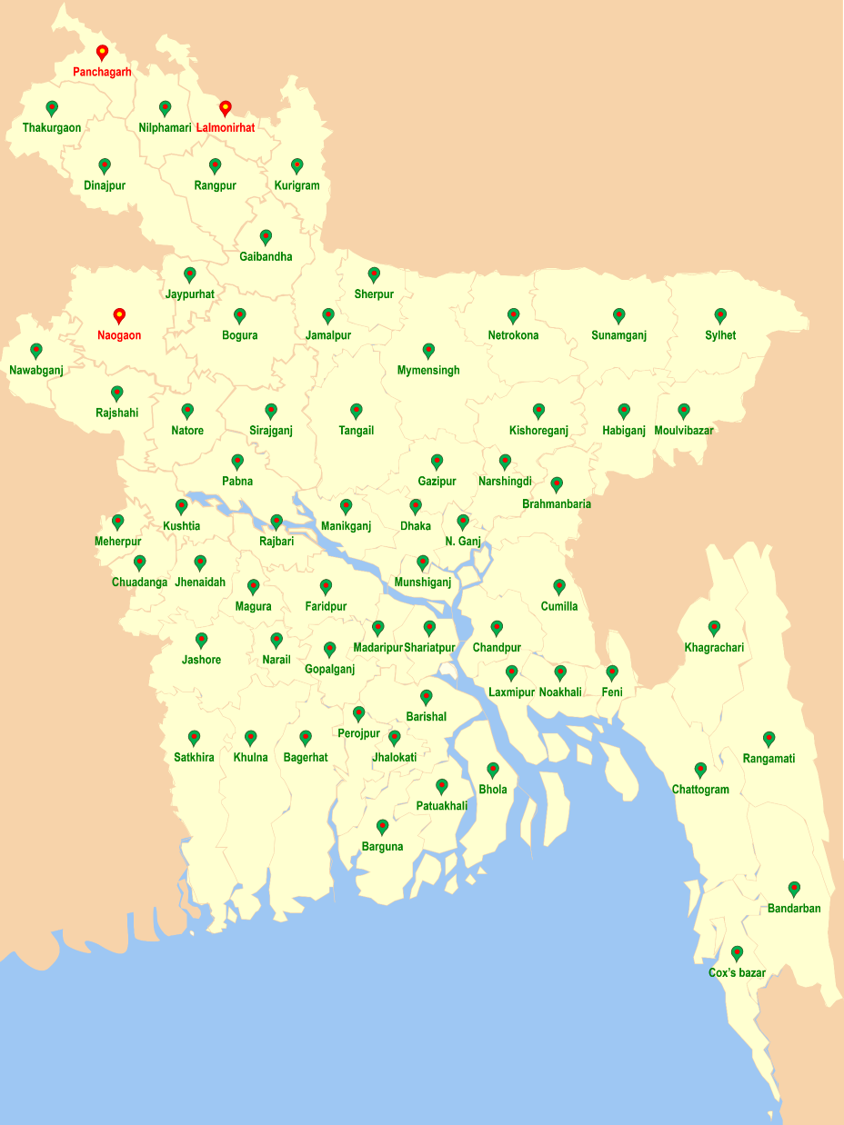

Supplement: Supplementary file 2 [file Image_2.TIF]

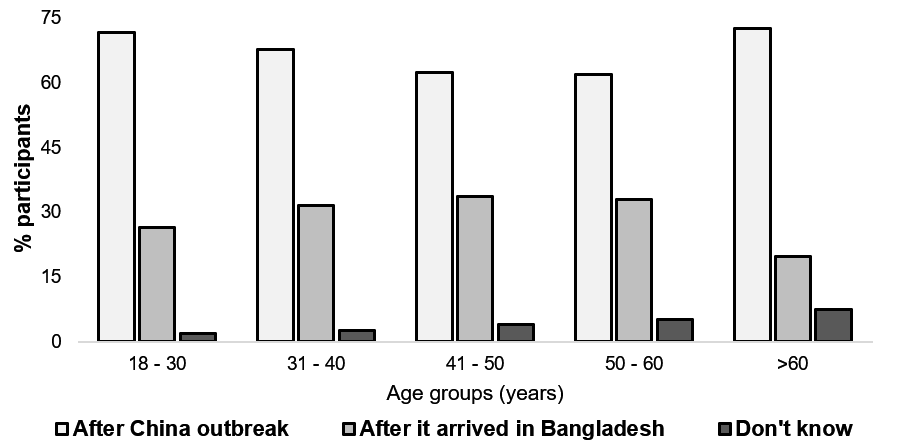

Supplement: Supplementary file 3 [file Image_3.TIF]

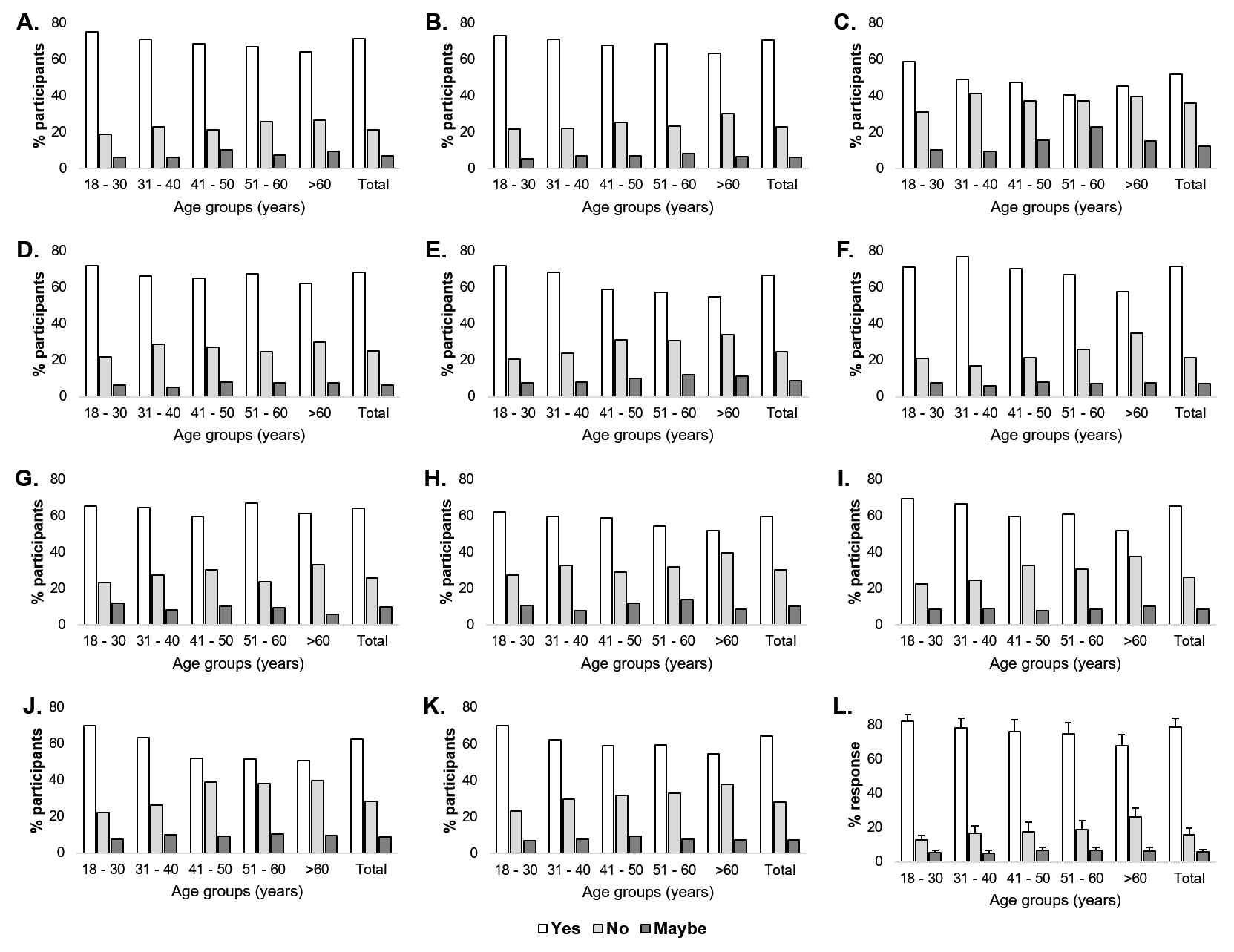

Supplement: Supplementary file 4 [file Image_4.TIF]
