# Supplementary material for: Women's Knowledge, Attitude, and Perceptions Toward COVID-19 in Lower-Middle-Income Countries: A Representative Cross-Sectional Study in Bangladesh
Source: Front Public Health. 2020 Nov 17;8:571689. doi: 10.3389/fpubh.2020.571689 (PMC7707120; doi:10.3389/fpubh.2020.571689)
Supplement: Supplementary file 5 [file Table_1.DOCX]

**S1 Table:** Participants’ response rate

| **Features** | **Value** |
| --- | --- |
| Total no. of individuals invited (n) | 3150 |
| Declined to participate (n, %) | 1246, 39.56% |
| Reasons for declining to participate (n, %) |  |
| No time to participate in the interview | 756, 60.67 |
| Not interested in participating | 346, 29.13 |
| Fear of deception, forgery | 144, 11.56 |
| Excluded due to incompleteness of questionnaire (n) | 35 |
| Questionnaires finally included in our analysis (n) | 1869 |
| Overall response rate (%) | 59.33 |

**S2 Table:** Clinical information of the participants

| **Clinical conditions (n, %)** | **Value  (n = 1869)** |
| --- | --- |
| Coronary complications |  |
| Present | 205, 10.96 |
| Not present | 1573, 84.17 |
| Do not know | 91, 4.87 |
| Frequent feverish syndrome |  |
| Present | 184, 9.84 |
| Not present | 1550, 82.93 |
| Maybe | 135, 7.23 |
| History of surgeries |  |
| Present | 387, 20.70 |
| Not present | 1482, 79.30 |
| Use of immunosuppressive drugs |  |
| Yes | 125, 6.69 |
| No | 1468, 78.55 |
| Do not know | 276, 14.76 |
| Pulmonological complications |  |
| Present | 234, 12.52 |
| Not present | 1472, 78.76 |
| Do not know | 163, 8.72 |
| Respiratory complications |  |
| Present | 195, 10.43 |
| Not present | 1558, 83.36 |
| Do not know | 116, 6.21 |
| Other major clinical conditions |  |
| Present | 260, 13.91 |
| Not present | 1609, 86.09 |
| Comorbid condition (>1 clinical conditions) |  |
| Present | 226, 12.09 |
| Not present | 1643, 87.91 |

**S3 Table:** Sources of knowledge about COVID-19 among the women (n = 1813) participated in this study

| **Source of knowledge** | **Value (n, %)** |
| --- | --- |
| TV | 919, 50.69 |
| Newspaper | 669, 36.9 |
| Internet | 1173, 64.7 |
| Public discussion | 526, 29.01 |
| Other |  |
| Family members | 390, 21.51 |
| Friends | 353, 19.47 |
| Relatives/acquaintances | 341, 18.8 |
| Radio | 166, 9.16 |
| Medical personnel | 102, 5.63 |
